# Bilayered negative-pressure wound therapy preventing leg incision morbidity in coronary artery bypass graft patients: A randomized controlled trial

**DOI:** 10.1097/MD.0000000000005925

**Published:** 2017-01-20

**Authors:** Yongchao Yu, Zhigang Song, Zhiyun Xu, Xiaofei Ye, Chunyu Xue, Junhui Li, Hongda Bi

**Affiliations:** a Department of Cardiac Surgery; b Department of Statistics, Faculty of Medical Services; c Department of Plastic Surgery, Changhai Hospital, Second Military Medical University, Shanghai 200433, China.

**Keywords:** bilayered negative pressure wound therapy (b-NPWT), coronary artery bypass graft (CABG), great saphenous veins, lymphorrhagia

## Abstract

**Backgrounds:**

The harvesting of great saphenous veins for coronary artery bypass graft (CABG) patients may result in significant complications, including lymphorrhagia, lymphoedema, incision infection, wound dehiscence, and skin flap necrosis. We investigated the function of a self-designed bilayered negative pressure wound therapy (b-NPWT) for reducing the above-mentioned complications using a clinical randomized controlled trial.

**Methods:**

A single-center, pilot randomized controlled trial was conducted. From December 2013 to March 2014, a total of 72 coronary heart disease patients (48 men and 24 women) received CABG therapy, with great saphenous veins were selected as grafts. Patients were equally randomized into a treatment and a control group. After the harvesting of the great saphenous veins and direct closure of the wound with sutures, b-NPWT was used for the thigh incision in the treatment group for 5 days (treatment thigh). Traditional surgical pads were applied to both the shank incisions of the treatment group patients (treatment shank) and the entire incisions of the control group (control thigh, control shank). Postoperative complications were recorded and statistically analyzed based on outcomes of thigh treatment, shank treatment, thigh control, and shank control groups.

**Results:**

The incidence rates of early complications, such as lymphorrhagia, lymphoedema, infection, wound dehiscence, and skin flap necrosis, of the vascular donor site in the thigh treatment group was significantly lower than those in the 3 other groups.

**Conclusions:**

The self-designed b-NPWT can effectively reduce postoperative complications, such as lymphedema, incision infection, wound dehiscence, and skin flap necrosis, in CABG patients who underwent great saphenous veins harvesting.

**Trial registration:**

ClinicalTrials.gov. The unique registration number is NCT02010996.

## 1 Introduction

Great saphenous veins are long, tough, and easily sutured, and are widely used as grafts in 98% of coronary artery bypass graft (CABG) operations.^[1]^ The harvesting of great saphenous veins in CABG patients may result in numerous complications, with reports of complication in 44% of patients.^[2]^ The rate of complications at the great saphenous veins harvest site is higher than noted at the chest incision.^[3]^ The harvesting of the great saphenous veins in CABG patients is associated with complications, including lymphedema, incision infection, wound dehiscence, and skin flap necrosis.^[4–6]^ The primary cause of these complications is lymphatic obstruction.^[7]^

Previously, we developed a bilayered negative pressure wound therapy (b-NPWT) for the treatment of prolonged, poorly healed postmastectomy seroma and achieved impressive results.^[8]^ In this study, we tested a similar system immediately following bypass surgery for preventing wound-related complications in the vascular donor site after harvesting the great saphenous veins.

## 2 Patients and methods

This study was conducted following the guidelines set by the Consolidated Standards of Reporting Trials.

### 2.1 Patients

The patient inclusion criteria included coronary heart disease with a transplant of more than 2 vascular bridges, thigh groin following the saphenous vein, and written informed consent. The patient exclusion criteria included reluctance to participate in clinical trials, injury, thigh operation history, chemotherapy before surgery, and known hypersensitivity to components of the surgical adhesive membrane. The patient details are given in Table 1.

**Table 1:**
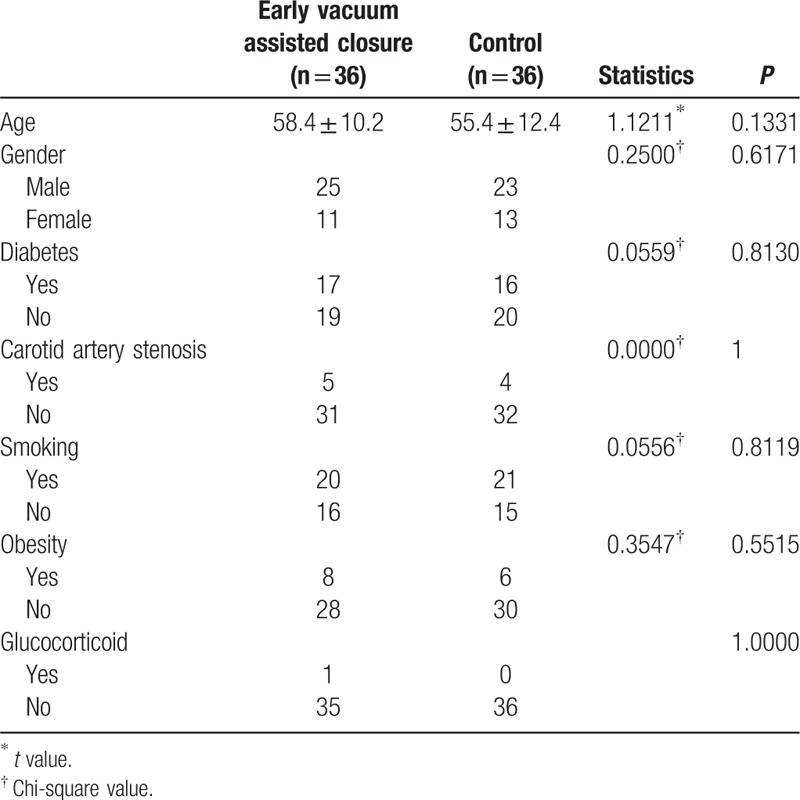
Baseline characteristics of the study population.

### 2.2 Study design

This single-center, randomized, open-label, standard-controlled study was conducted to evaluate the efficacy and safety of early b-NPWT application for preventing postoperative complications and promoting wound healing after stripping the saphenous vein in coronary bypass.

This study was performed in Changhai Hospital in Shanghai, China. Changhai Hospital, also known as the First Affiliated Hospital of the Second Military Medical University, is one of the largest hospitals in Shanghai. Patients who underwent stripping of the saphenous vein during coronary bypass between December 2013 and April 2014 were enrolled in this study.

The Institutional Review Board of Changhai Hospital approved this study. Written informed consent was obtained from all the patients before enrolment. This study was done in accordance with the ethics principles of the Declaration of Helsinki and was consistent with good clinical practices and applicable laws and regulations. Moreover, this trial is registered at ClinicalTrials.gov under number NCT02010996.

### 2.3 Randomization and masking

The randomization sequence was computer generated, and the sequence code was placed in an opaque sealed envelope, which was opened by a physician at the time of the enrolment of a particular patient. The trial was an open-label clinical trial.

### 2.4 Surgical procedure

Patients underwent a classical CABG operation with cardiopulmonary bypass. We incised the skin and subcutaneous tissue according to the anatomy of the great saphenous veins from groin to ankle with several intervals to avoid dysfunction of the joint caused by a linear scar. The great saphenous veins were peeled, branches were ligated, and grafts were finally harvested. All operations were performed by the same Surgical group and Anesthesia group. All patients were treated in the same operation room and intensive care unit. A second generation cephalosporins was given half an hour before the operation to prevent infection, and discontinued 72 hours after surgery. We closely monitored the blood sugar of patients during the perioperative period. The postoperative blood glucose was well controlled well in all patients with hypoglycemic therapy.

### 2.5 Implementation of b-NPWT

**Figure 1:**
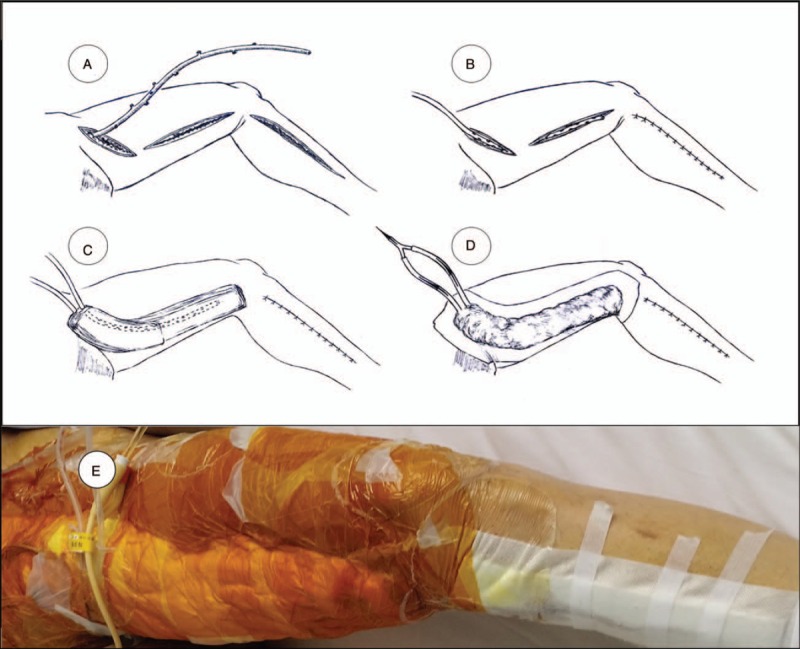
Diagrammatic sketch of b-NPWT implementation. A: Harvest of a great saphenous vein through an interrupted incision in the lower limb. B: Insertion of a drainage tube into the bottom of the wound. C: After closure of the incision with sutures, the skin surface of the thigh was protected with multiple vaseline gauzes in which the second drainage tube was inserted. D: The gauze was fixed and compressed on the skin with adhesives tape, and 2 tubes were connected to a negative pressure source (−120 mm Hg) followed by sustained vacuum suction. E: Gloss view of the b-NPWT in a patient. b-NPWT = bilayered negative pressure wound therapy.

The b-NPWT developed by our department was manually implemented as follows: a drainage tube was inserted in the bottom of the wound after harvesting of the great saphenous veins (Fig. 1A, B). Sterilized gauze pads (3.5 cm thick) were placed on the surface of the skin after the incision was directly closed by suturing. The gauze coverage was 2.5 to 3.0 cm over the incision line in width bilaterally. A second drainage tube was placed over the gauze pad (Fig. 1C). After another 3 to 5 layers of gauze were applied over the second drainage tube, and the system carefully sealed with the skin using an adhesive film. Both tubes (inside the cavity and inside gauzes) were connected to a negative pressure source (−120 mm Hg), followed by application of a constant vacuum suction.

The wound in the control group was directly closed by suturing without inserted a drainage tube.

### 2.6 Study protocol

The primary objective of the study was to compare the incidence of thigh complications between the treatment and control groups. The second objective was to compare the incidence rates of complications in the thigh and shank between the treatment and control groups.

A sample size of 72 patients allocated at a 1:1 ratio was planned at a 0.05 significance level (90% power), with the assumption that the percentages of the true incidence of complications in the 2 groups were 25% and 1%, respectively. A data safety monitoring board was not used in this study because no interim analyses were undertaken.

Patients were enrolled immediately after harvesting of saphenous vein during coronary bypass. The first patient visit occurred on December 4, 2013; the datalock for the primary analysis occurred on May 6, 2014; and the final datalock for analysis occurred on May 21, 2014.

### 2.7 Statistical analysis

Continuous data in normal distribution was expressed as mean ± standard deviation. A comparison of continuous data between two groups was conducted using Student *t* test. Chi-square test or Fisher exact test was used for categorical data. Fisher exact test was performed where the expected value for any of the contingency table was less than 1. Continuous correction for Chi-square test was performed where the minimum expected value for the contingency table was between 1 and 5. We used statistic analysis system (SAS) versions 9.3 (SAS institute, Cary,NC) for statistical analyses. A value of *P* < 0.05 was considered statistically significant.

## 3 Results

A total of 72 patients who underwent coronary artery bypass were enrolled in this study. The baseline parameters were similar for the 2 groups (Table 1). No statistically significant difference was observed for age, sex, diabetes history, carotid artery stenosis, smoking, obesity, or use of glucocorticoids.

### 3.1 Complications

#### 3.1.1 Early total body complications

Neither death nor new cardiovascular event was observed in the 2 groups. All patients who underwent coronary bypass were prescribed aspirin for anticoagulant therapy the day after surgery. After the chest tube was removed, clopidogrel anticoagulation was introduced. No deep vein thrombosis occurred in either groups.

#### 3.1.2 Early vascular donor site complications

Early vascular donor site complications included lymphoedema, incision infection, wound dehiscence, and skin flap necrosis. Lymphoedema of the thigh or shank was determined by a 2 cm difference between the operated and non-operated sides. The thigh perimeter was measured around the midpoint between the anterior superior iliac spine and the patella. The shank perimeter was measured around the midpoint between the patella and the ankle.

The incidence of early vascular donor site complications in the thigh treatment group was significantly lower than that in the thigh control group (*χ*^2^ = 15.7500, *P* < 0.0001, Table 2). In addition, the incidence of early vascular donor site complications in the thigh control group was significantly higher than that in the shank control group (*χ*^2^ = 6.9231, *P* = 0.0085). However, no significant difference was observed between the thigh treatment and shank treatment group (*χ*^2^ = 0.8597, *P* = 0.3538).

**Table 2:**
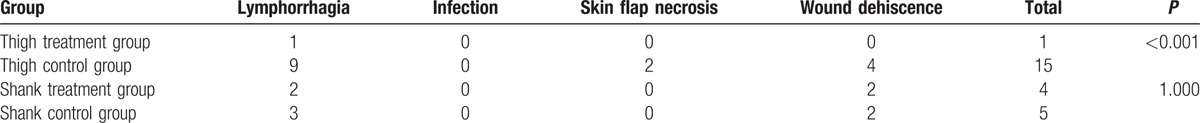
Description of early vascular donor site complications.

#### 3.1.3 Long-term complications

The long-term complication was characterized by chronic lymphoedema. We conducted a follow-up with the patients 3 months after they left the hospital. The diagnosis criterion of chronic lymphoedema was a 2 cm difference between the operated and non-operated sides. The thigh perimeter was measured around the midpoint between the anterior superior iliac spine and the patella. The shank perimeter was measured around the midpoint between the patella and the ankle.

The incidence of long-term complications in the thigh treatment group was significantly lower than that in the thigh control group (*χ*^2^ = 6.8906, *P* = 0.0087, Table 3). In addition, the incidence of long-term complications in the thigh control group was significantly higher than that in the shank control group (*χ*^2^ = 4.1806, *P* = 0.0409). However, no significant difference was observed between the thigh treatment and shank control groups (*P* = 1).

**Table 3:**
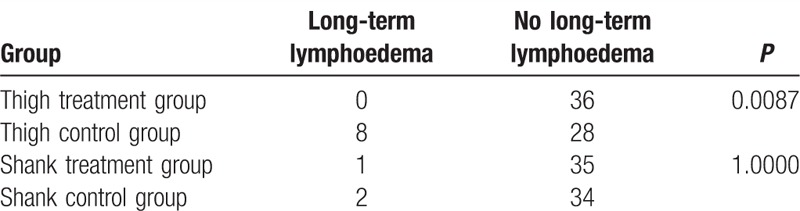
Description of long-term complications.

### 3.2 Typical cases

Figure 2.

**Figure 2:**
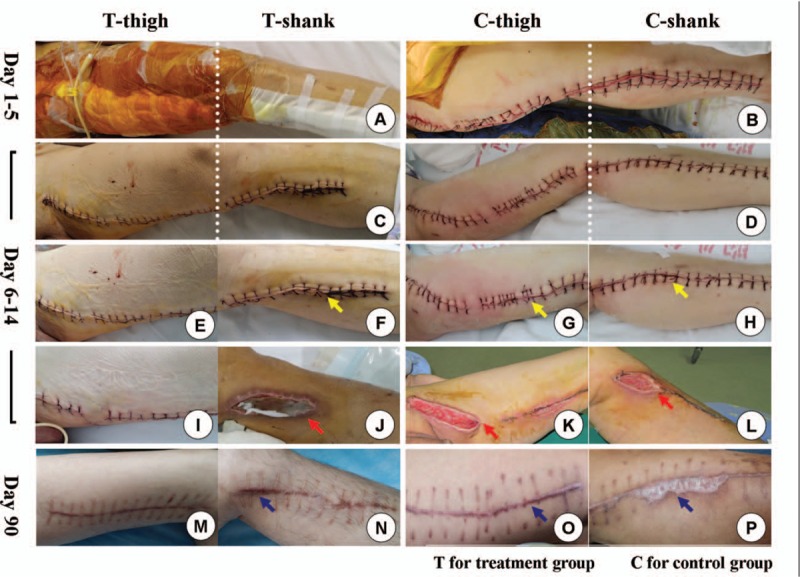
Typical cases and long-term follow up. The b-NPWT treatment was immediately implemented covering incision in the thigh of treatment group (A), and removed in day 5 (C), while traditional surgical pad was used to protect the wound in control group in the same period (B, D). Skin and subcutaneous tissue edema (F, G, and H) occurred in T-shank, C-thigh, and C-shank (yellow arrow) without exception; in contrast, local skin remained in contractive appearance in T-thigh for several days after the cessation of b-NPWT (E). Significant complications occurred in T-shank, C-thigh, and C-shank (J, K, L, red arrow), including lymphorrhagia, incision infection, and wound dehiseence, while the incision healed unevenly in T-thigh (I) without hypertrophic scar (M) that usually formed in the control site (N, O, P) during 3 months’ follow up. C-shank = control shank, C-thigh = control thigh, T-shank = treatment shank, T-thigh = treatment thigh.

## 4 Discussion

Although the direct incision and stripping of the great saphenous vein may lead to leg incision complications, such as postoperative lymphatic leakage, it remains the most widely accepted technique for CABG.^[9]^ To our knowledge, this is the first attempt to prevent postoperative wound related complications with a novel method based on b-NPWT.

b-NPWT is widely promoted as a treatment for full thickness soft tissue defects.^[10,11]^ b-NPWT modulates blood infusion, stimulates vascularization, promotes regional granulation, and eventually aids in wound healing.^[12–14]^ However, the “negative pressure” generated by suction may only penetrate the limited superficial tissue,^[15,16]^ and a positive force exists between the local tissue and NPWT media (foam).^[17–19]^ This direct force may be utilized to fasten a free skin graft to the wound bed and achieve wound healing.^[20,21]^ Moreover, lymph-vessel regeneration and lymphatic reconstruction were also accelerated by NPWT in chronic wounds.^[22,23]^ These findings indicate a potential role for NPWT in clinical problems caused by lymphatic disturbance, such as lymphocysts and lymphorrhagia.

Lymphorrhagia is a common surgical complication following various operations.^[24]^ Regional lymph node dissection, such as axillary lymph node dissection and inguinal lymph node dissection, has long been accompanied by a high complication rate and is characterized by aggravated fluid accumulation.^[25–29]^ In 44% of CABG patients, lymphorrhagia may occur in the saphenous vein donor site, causing a prolonged hospital stay.^[2]^ Moreover, lymphorrhagia is usually the initial cause of other severe wound related complications, including wound break down, infection, and skin flap necrosis.^[30–33]^ Although the definite causes of lymphorrhagia remain unclear, it is a common practice to apply dynamic local pressure and efficient drainage to achieve wound healing. Our previous study showed that a manually implanted bilayered NPWT system was effective for a long-term poorly healed postmastectomy seroma.^[8]^ This system produced a dynamic constant local pressure and constant fluid discharging simultaneously, steadily minimizing the regional dead space and promoting wound healing. This led us to test a similar system for complication control in the saphenous vein donor site after bypass surgery using a single center randomized controlled trial (RCT).

This short pilot study successfully provided important support for postoperative morbidity prevention after bypass surgery by the application of b-NPWT. Compared with the control group, wound related complications were significantly reduced in the thighs of the treatment group. Furthermore, control was also preset in the shank of the treatment group since the general risk of complications is much higher in the groin region than in the lower limb due to lymph circulation. Several patients (4/32) who developed severe complications, including lymphorrhagia and wound dehiscence, in the shank of the treatment group, and the thigh incision healed uneventfully under b-NPWT. This statistically significant difference in the same patient who received the same medical care provides meaningful support for this therapy. We find the protective effect of b-NPWT only occurs within the suction coverage area, and supports our hypothesized mechanism of b-NPWT, that it mainly relies on the physical pressure and discharge of abnormal fluid.

An important controversy regarding NPWT is the lack of high-quality research evidence regarding its clinical and cost effectiveness.^[34]^ This clinical trial demonstrated the efficacy of a novel modification of NPWT in problematic wound protection. Moreover, this method does not rely on hydrophobic foam media, which is required in commercial NPWT system.^[35]^ All components of b-NPWT were clinically available, and such a treatment would not increase the economic burden of the patients. Because wound complications only occurred in a fraction of total patients and were notable to clinicians for signs of abnormal fluid seeping and inflammation, we propose this method for patients after harvesting of the great saphenous veins because previously it has been used to successfully treat various complications following similar incisions.

## 5 Conclusions

This study showed that a self-designed b-NPWT was a helpful treatment for reducing leg incision complications in CABG patients. More cases should be collected or a multiple-center study needs be initiated in the future to further evaluate the best indications of this method for other similar problems.
